# Supplementary figures and images for: SNX27–Retromer directly binds ESCPE-1 to transfer cargo proteins during endosomal recycling
Source: PLoS Biol. 2022 Apr 13;20(4):e3001601. doi: 10.1371/journal.pbio.3001601 (PMC9038204; doi:10.1371/journal.pbio.3001601)

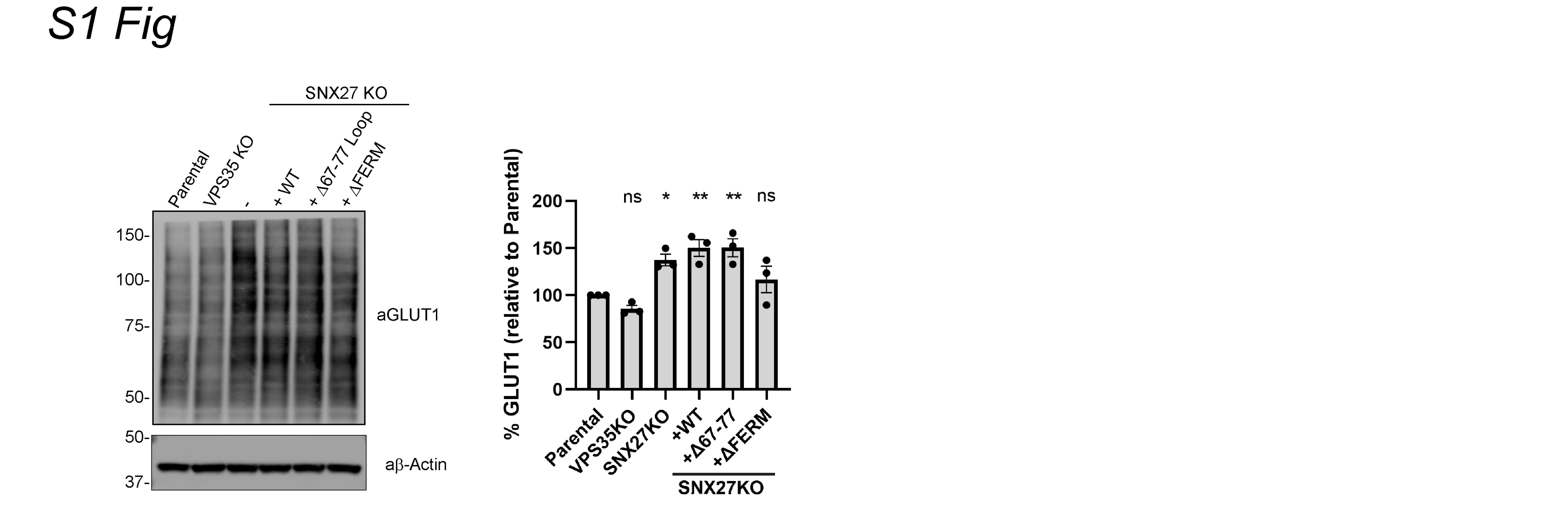

Supplement: S1 Fig — The blot is representative of 3 independent assays. Molecular masses are given in kilodaltons. Bars, error bars, and symbols represent the mean, SEM, and individual data points, respectively. *P < 0.05, **P < 0.01, ***P < 0.001, ****P < 0.0001, ns = not significant. The data underlying the graphs shown in the figure can be found in S1 Data. KO, knockout; SNX27, sorting nexin-27; WT, wild-type. (TIF) [file pbio.3001601.s001.tif]

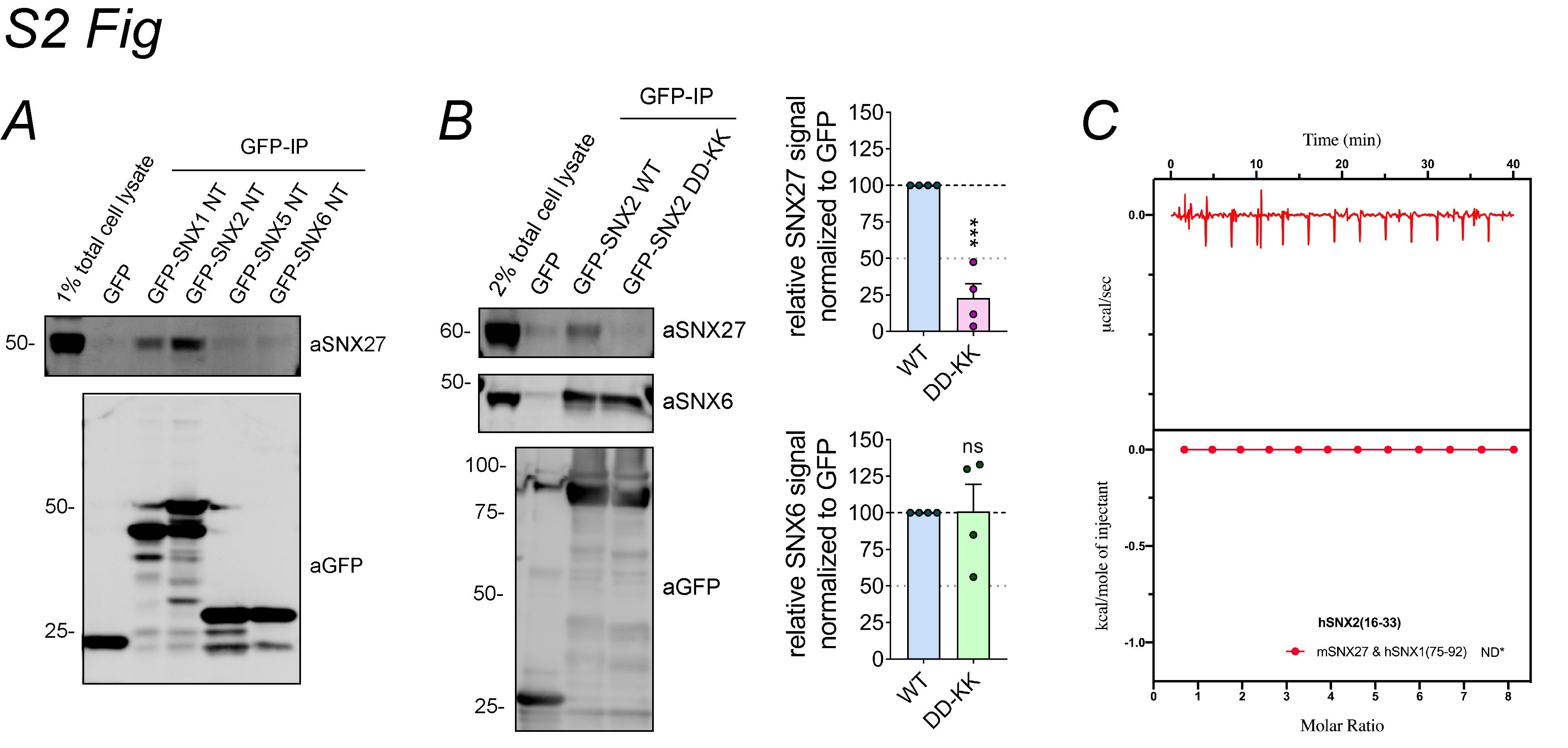

Supplement: S2 Fig — (A) Co-immunoprecipitation of GFP-tagged extended amino-terminal regions (NT) of SNX1 and SNX2 alongside the GFP-tagged short amino-terminal regions of SNX5 and SNX6 expressed in HEK293T cells. The cell lysates were subjected to GFP trap–based immunoprecipitation and the immunoprecipitates were blotted for SNX27 and GFP. The blot is representative of 3 independent GFP traps. (B) Co-immunoprecipitation of GFP-tagged SNX2 WT or SNX2 KK (D27K, D72K) expressed in HEK293T cells. The cell lysates were subjected to GFP trap–based immunoprecipitation and the immunoprecipitates were blotted for SNX27, SNX6, and GFP. The blot is representative of 4 independent GFP traps. (C) Competitive ITC assay of SNX216-33 and mSNX27FL preincubated with 2-fold molar excess of SNX175-92 peptide showing that binding is blocked by the competing SNX175-92 peptide. Molecular masses are given in kilodaltons. Bars, error bars, and symbols represent the mean, SEM, and individual data points, respectively. *P < 0.05, **P < 0.01, ***P < 0.001, ****P < 0.0001, ns = not significant. The data underlying the graphs shown in the figure can be found in S1 Data. SNX1, sorting nexin-1; SNX5, sorting nexin-5; SNX6, sorting nexin-6; SNX27, sorting nexin-27; WT, wild-type. (TIF) [file pbio.3001601.s002.tif]

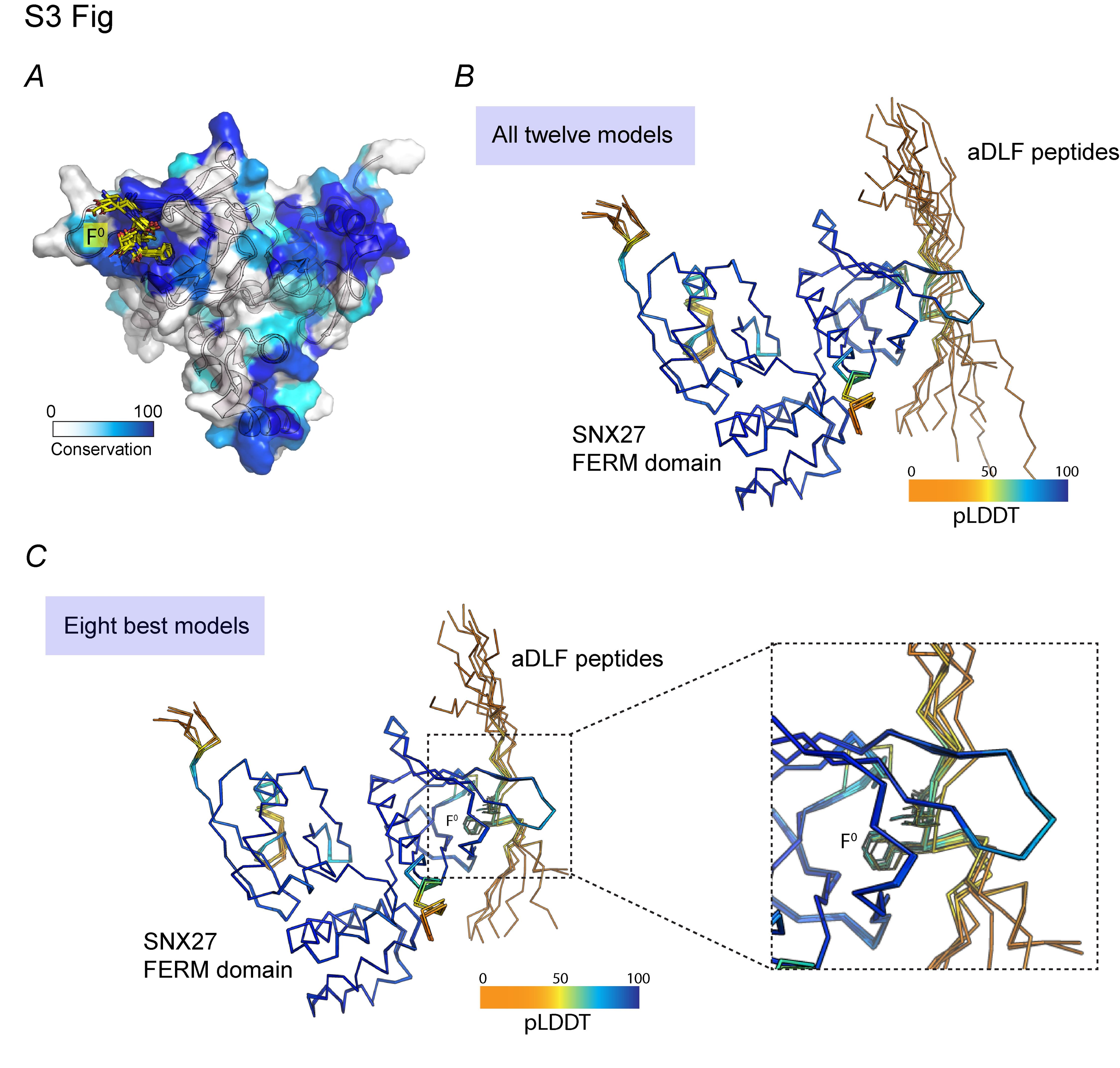

Supplement: S3 Fig — (A) SNX27 surface colored according to sequence conservation. The peptides are predicted to bind to a highly conserved pocket in SNX27. (B, C) As described in methods, 12 models of SNX27 were generated in total, consisting of 3 models each of SNX27 in association with the 4 aDLF sequences from human SNX1 and SNX2. B shows the overlay of all 12 models in Cα trace representation colored by the pLDDT score. The pLDDT is a per-residue confidence score between 0 (lowest confidence) and 100 (highest confidence). The majority of the SNX27 structures show very high pLDDT scores and overlay with very high precision. C shows only the best 8 models where the core aDLF sequences have identical binding conformations. aDLF, acidic-Asp-Leu-Phe; pLDDT, predicted Local Distance Difference Test; SNX1, sorting nexin-1; SNX2, sorting nexin-2; SNX27, sorting nexin-27. (TIF) [file pbio.3001601.s003.tif]

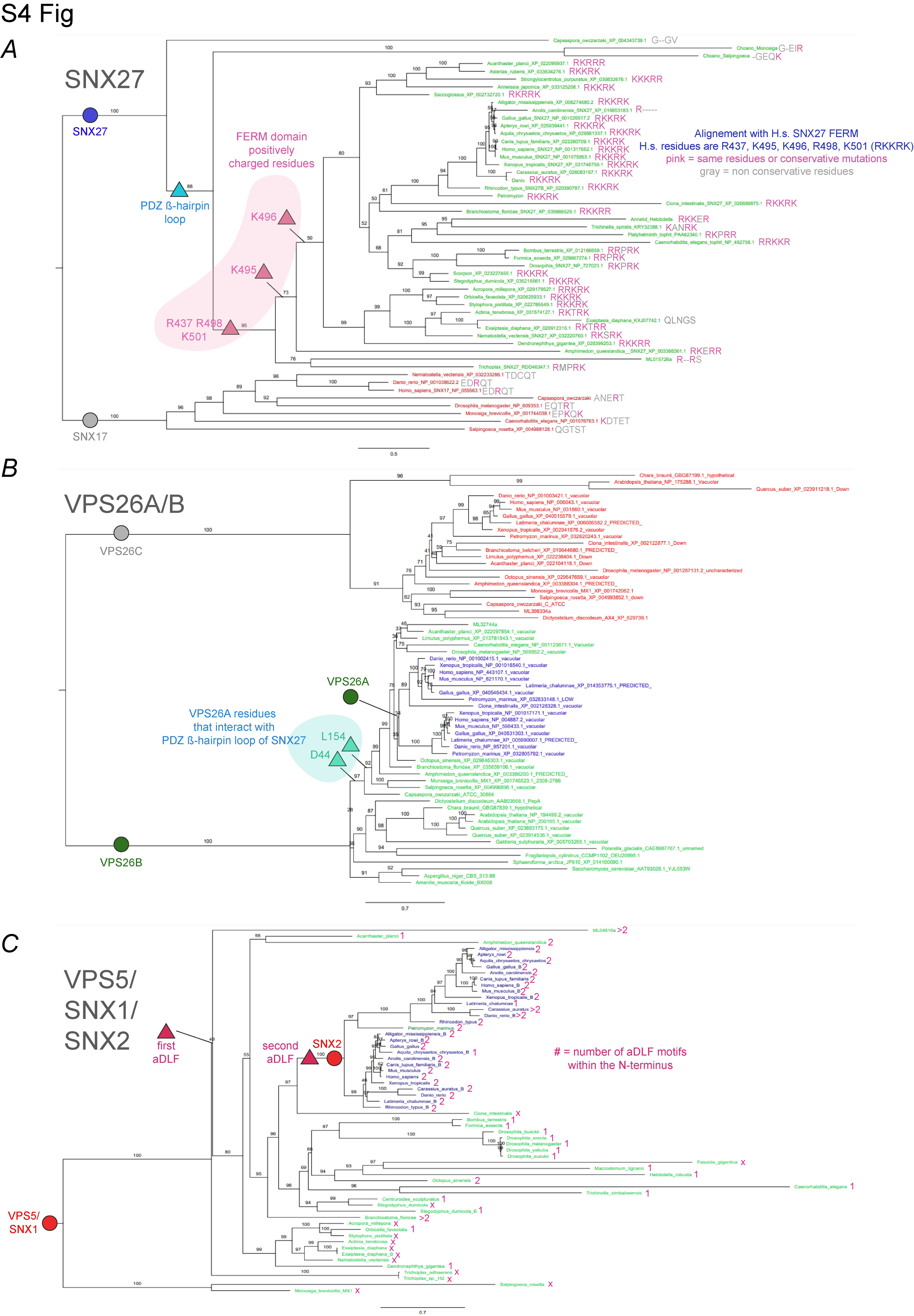

Supplement: S4 Fig — Maximum likelihood phylogenies of (A) SNX27, (B) VPS26A/B and (C) VPS5/SNX1/SNX2. (A) SNX27 (green) with SNX17 (red) used as an outgroup with representatives across Filozoa. Amino acids aligned with Homo sapiens in positions 437, 495, 496, 498, and 501 are written next to the species name. (B) VPS26A, VPS26B, and VPS26C, with VPS26C in red and VPS26B in green; the duplication event occurring in the ancestral vertebrate is in blue. Interestingly, we also find another independent duplication of VPS26B in plants evidenced by Arabidopsis thaliana and Quercus suber in this tree. (C) The SNX1 sequences across choanozoans, with the duplication in vertebrates in blue. The number of aDLF motifs in the amino terminus is written next to the species name. Phylogenies were inferred under the best-fit model as determined by BIC. Branch support values are percentages based on 10,000 ultrafast bootstraps. aDLF, acidic-Asp-Leu-Phe; BIC, Bayesian information criterion; SNX1, sorting nexin-1; SNX27, sorting nexin-27. (TIF) [file pbio.3001601.s004.tif]
