# Supplementary material for: SNX27–Retromer directly binds ESCPE-1 to transfer cargo proteins during endosomal recycling
Source: PLoS Biol. 2022 Apr 13;20(4):e3001601. doi: 10.1371/journal.pbio.3001601 (PMC9038204; doi:10.1371/journal.pbio.3001601)
Supplement: S1 Table — ITC, isothermal titration calorimetry. (PDF) [file pbio.3001601.s005.pdf]

**Supplementary Table 1. Thermodynamic parameters for the binding of SNX1/SNX2 with SNX27 by ITC.**

| Syringe | Cell | $K_d$ (μM) | $\Delta H$ (kcal/mol) | $\Delta G$ (kcal/mol) | $-T\Delta S$ (kcal/mol) |
|---|---|---|---|---|---|
| **hSNX27$_{FERM}$ against mSNX1 proteins** | | | | | |
| hSNX27$_{FERM}$ | mSNX1$_{1-139}$ | 39.10 ± 0.42 | -6.93 ± 0.01 | -6.02 ± 0.01 | -0.92 ± 0.02 |
| hSNX27$_{FERM}$ | mSNX1$_{FL}$ | 41.00 ± 1.82 | -6.23 ± 3.11 | -5.99 ± 0.02 | 0.24 ± 3.10 |
| **hSNX1/2 peptides against mSNX27$_{FL}$** | | | | | |
| hSNX1$_{35-51}$ | mSNX27$_{FL}$ | 33.43 ± 1.89 | -1.72 ± 0.65 | -6.11 ± 0.04 | -4.39 ± 0.64 |
| hSNX1$_{35-51}$ (D45K) | mSNX27$_{FL}$ | No binding detected | | | |
| hSNX1$_{75-92}$ | mSNX27$_{FL}$ | 13.37 ± 0.75 | -1.98 ± 0.18 | -6.65 ± 0.03 | -4.68 ± 0.17 |
| hSNX2$_{16-33}$ | mSNX27$_{FL}$ | 37.8 ± 1.44 | -0.85 ± 0.30 | -6.04 ± 0.02 | -5.19 ± 0.29 |
| hSNX2$_{16-33}$ (DLF-SSS) | mSNX27$_{FL}$ | No binding detected | | | |
| hSNX2$_{62-82}$ | mSNX27$_{FL}$ | 22.63 ± 1.89 | -1.85 ± 0.43 | -6.34 ± 0.05 | -4.49 ± 0.43 |
| **Competitive ITC assay of hSNX2$_{16-33}$ and hSNX1$_{75-92}$ with mSNX27$_{FL}$** | | | | | |
| hSNX2$_{16-33}$ | mSNX27$_{FL}$ & hSNX1$_{75-92}$ | No binding detected | | | |
| hSNX1$_{75-92}$ | mSNX27$_{FL}$ & hSNX2$_{16-33}$ | 30.35 ± 1.06 | -2.56 ± 0.14 | -6.17 ± 0.02 | -3.61 ± 0.12 |
| **hSNX1 peptide 75-92 against hSNX27$_{FL}$ WT and mutants** | | | | | |
| hSNX1$_{75-92}$ | hSNX27$_{FL}$ | 12.77 ± 0.47 | -1.65 ± 0.54 | -6.68 ± 0.03 | -5.03 ± 0.54 |
| hSNX1$_{75-92}$ | hSNX27$_{FL}$ K495D | No binding detected | | | |
| hSNX1$_{75-92}$ | hSNX27$_{FL}$ K496D | 56.40 ± 2.83 | -1.30 ± 1.07 | -5.80 ± 0.03 | -4.50 ± 1.04 |
| hSNX1$_{75-92}$ | hSNX27$_{FL}$ R498D | No binding detected | | | |
| hSNX1$_{75-92}$ | hSNX27$_{FL}$ K501D | No binding detected | | | |
