# Supplementary material for: SNX27–Retromer directly binds ESCPE-1 to transfer cargo proteins during endosomal recycling
Source: PLoS Biol. 2022 Apr 13;20(4):e3001601. doi: 10.1371/journal.pbio.3001601 (PMC9038204; doi:10.1371/journal.pbio.3001601)

Figure 1A

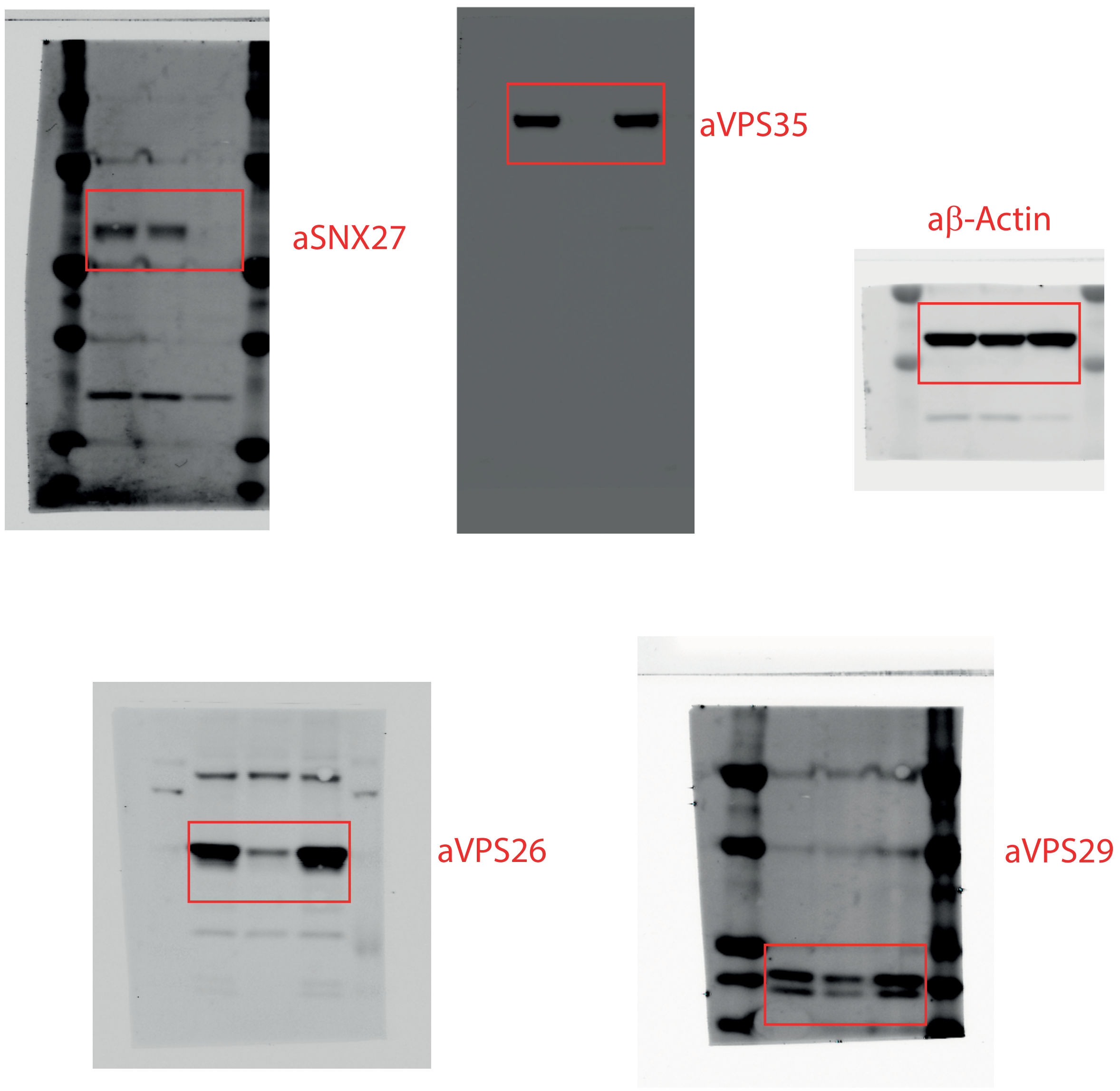

Supplementary Figure 1

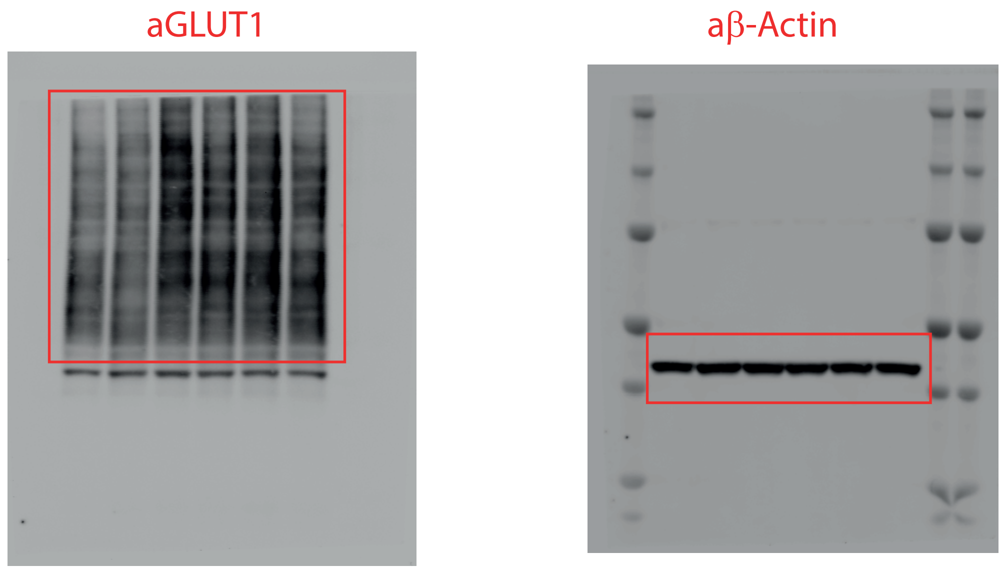

Figure 2C

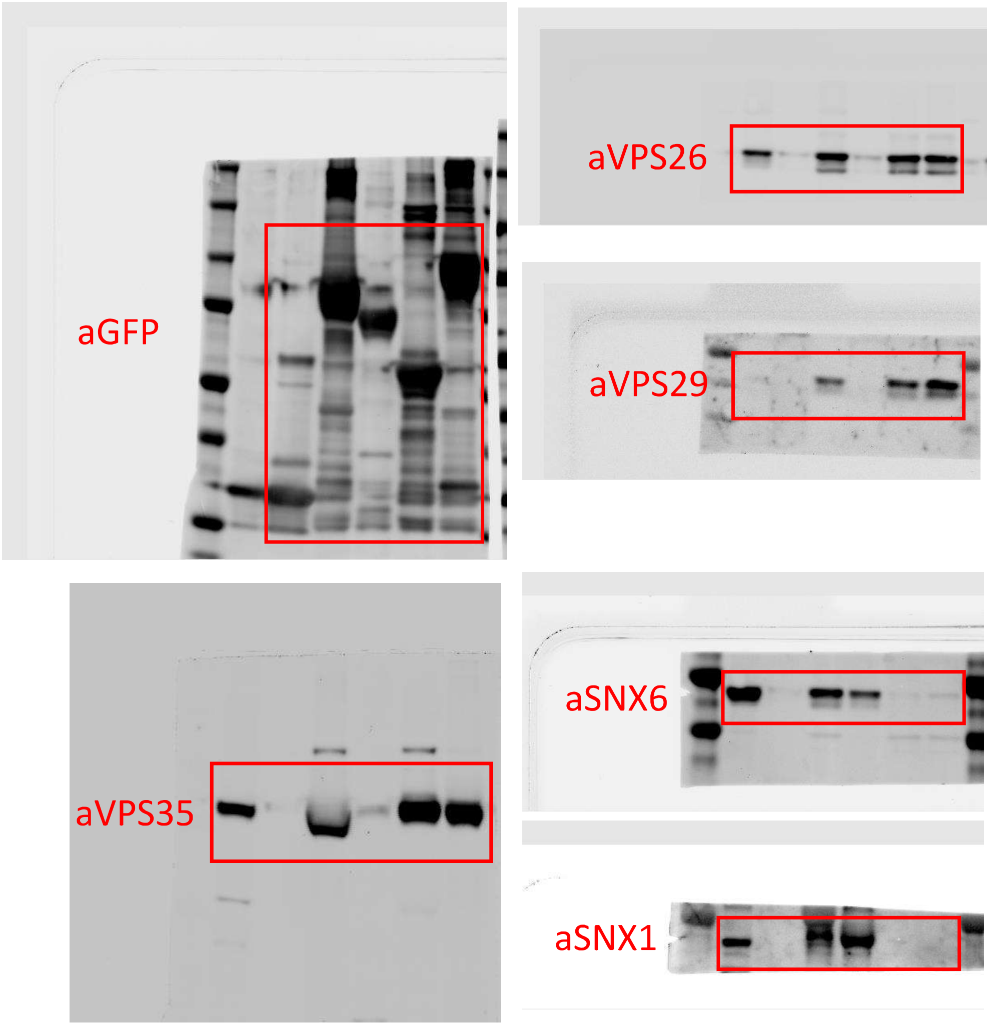

Figure 2D

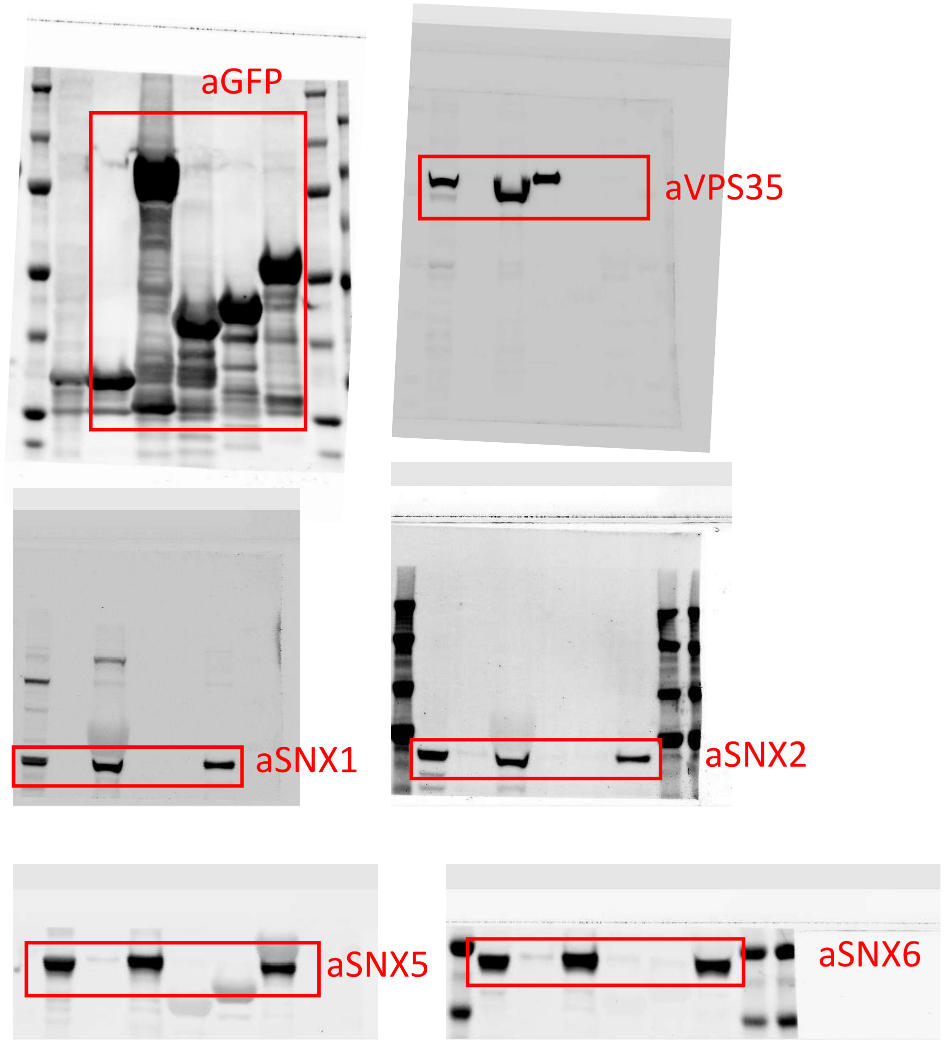

Figure 2E

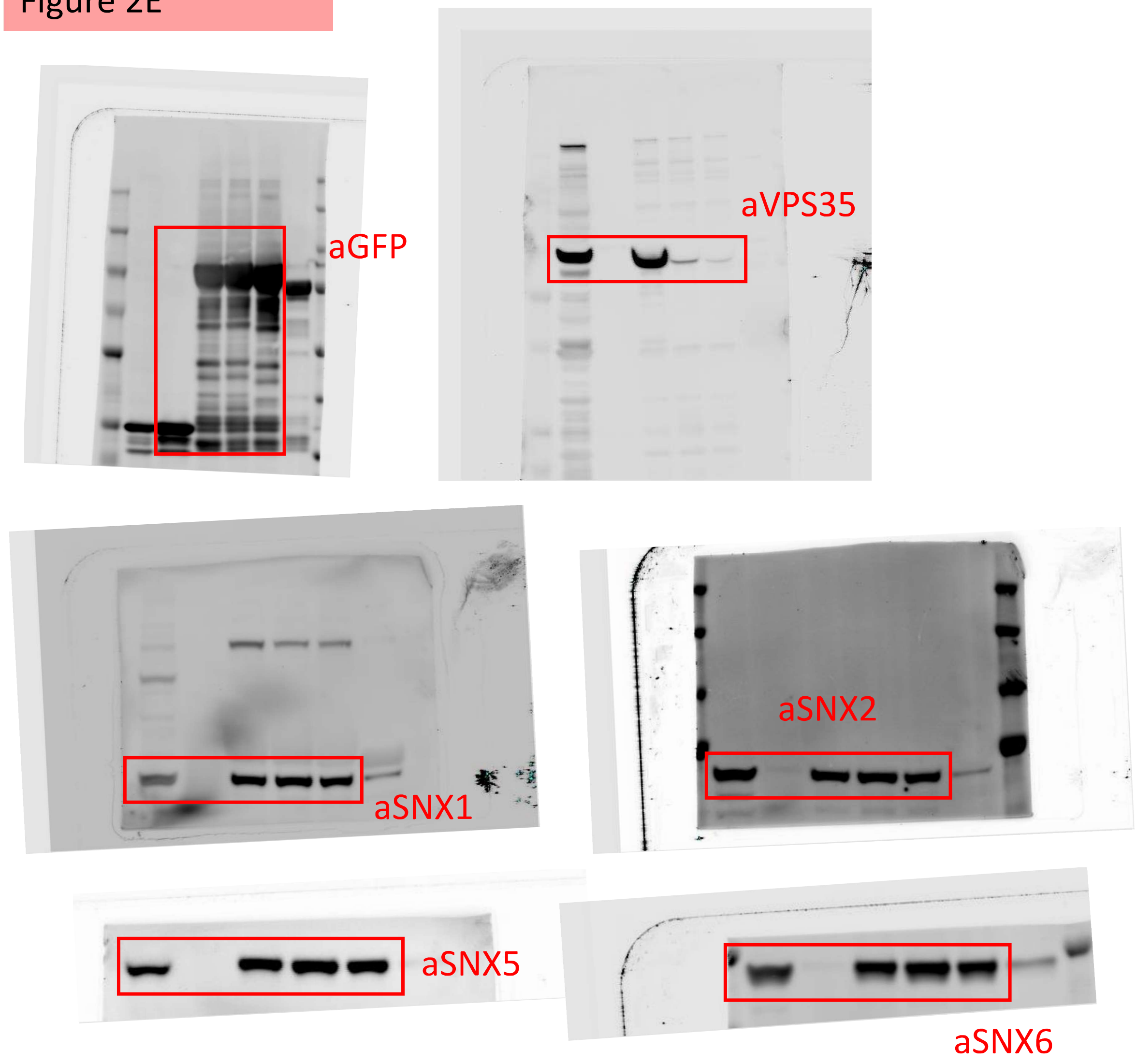

Figure 2F

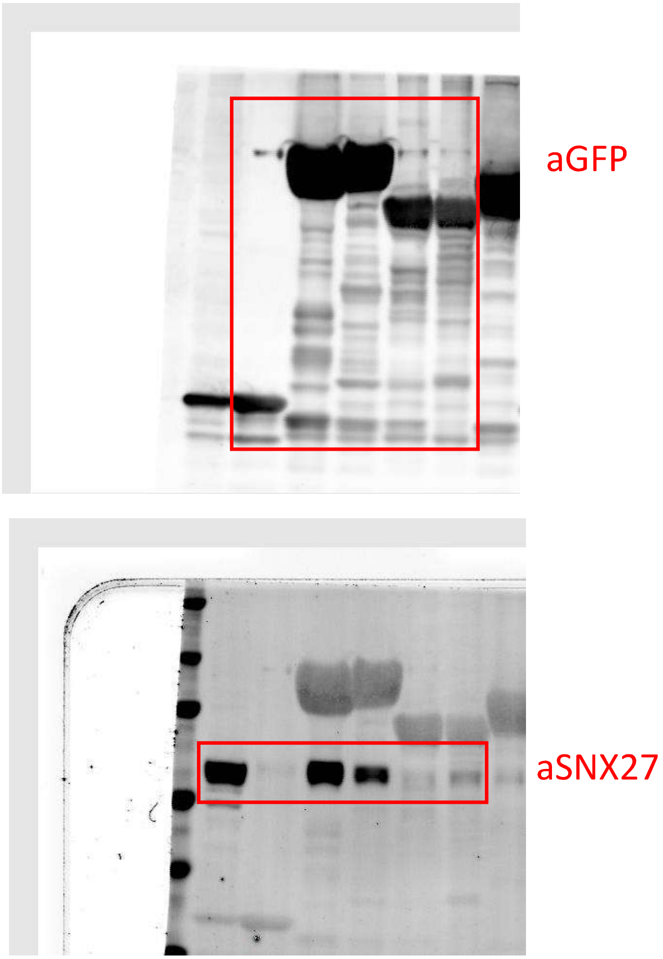

Figure 2G

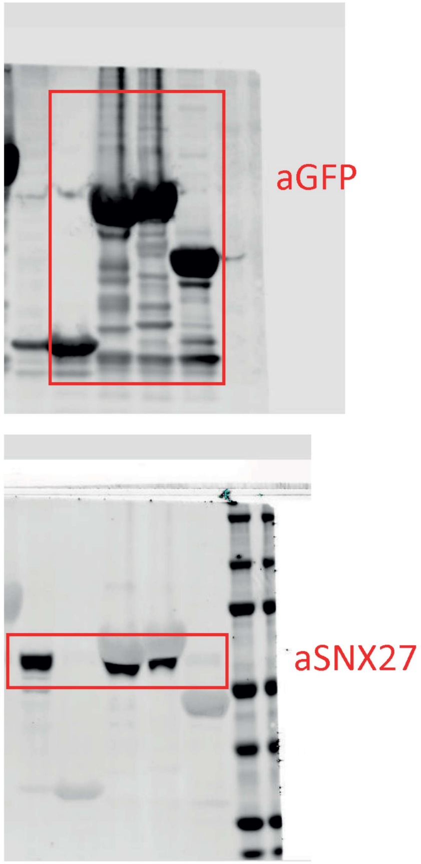

Figure 3A

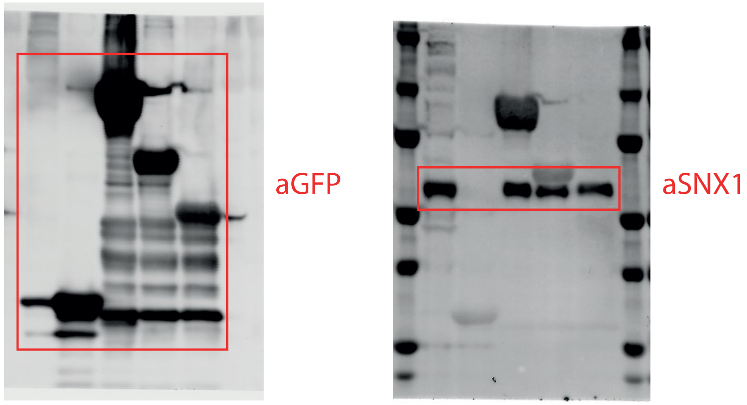

Supplementary Figure 2A

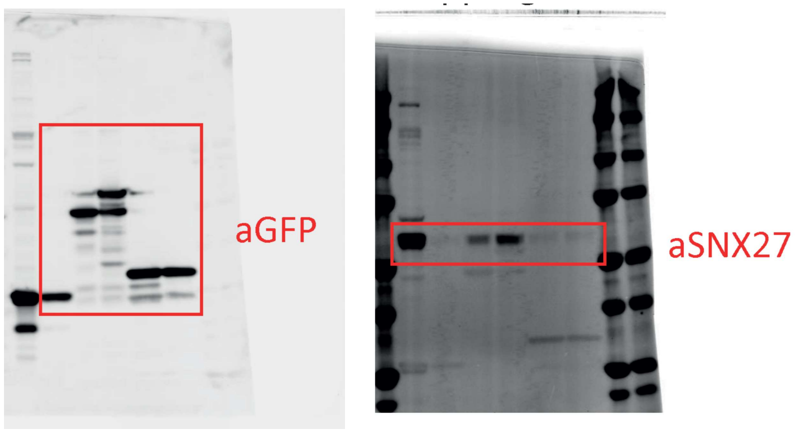

Supplementary Figure 2B

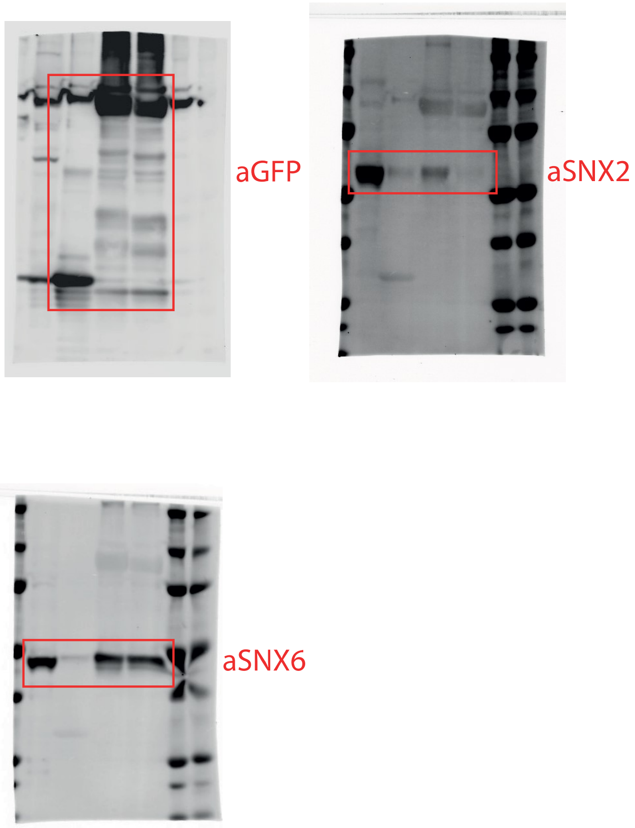

Figure 3C

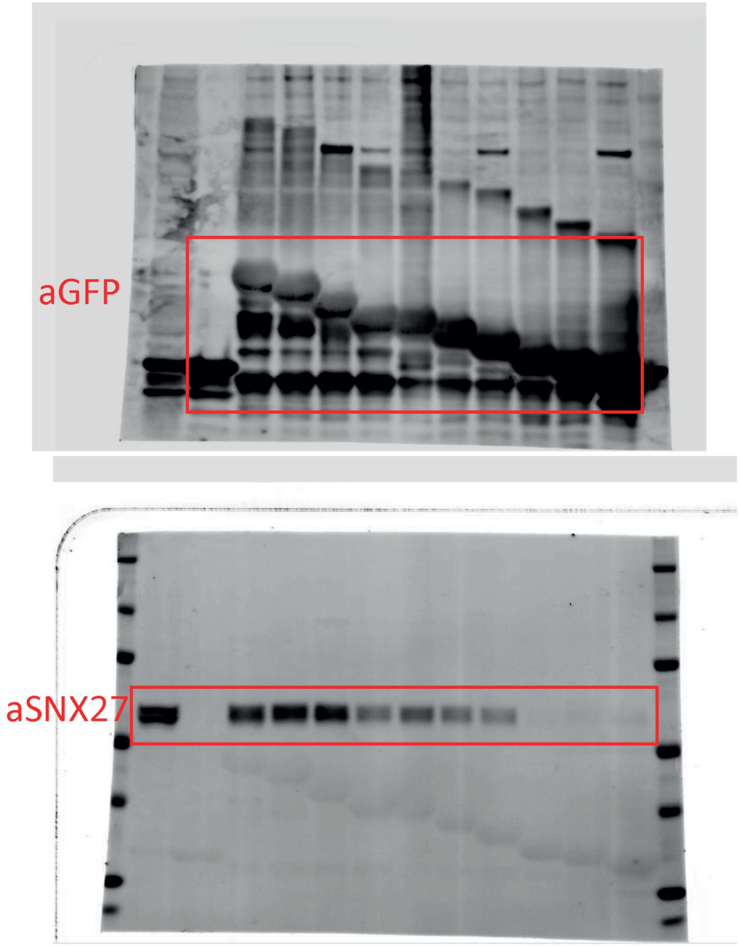

Figure 3F

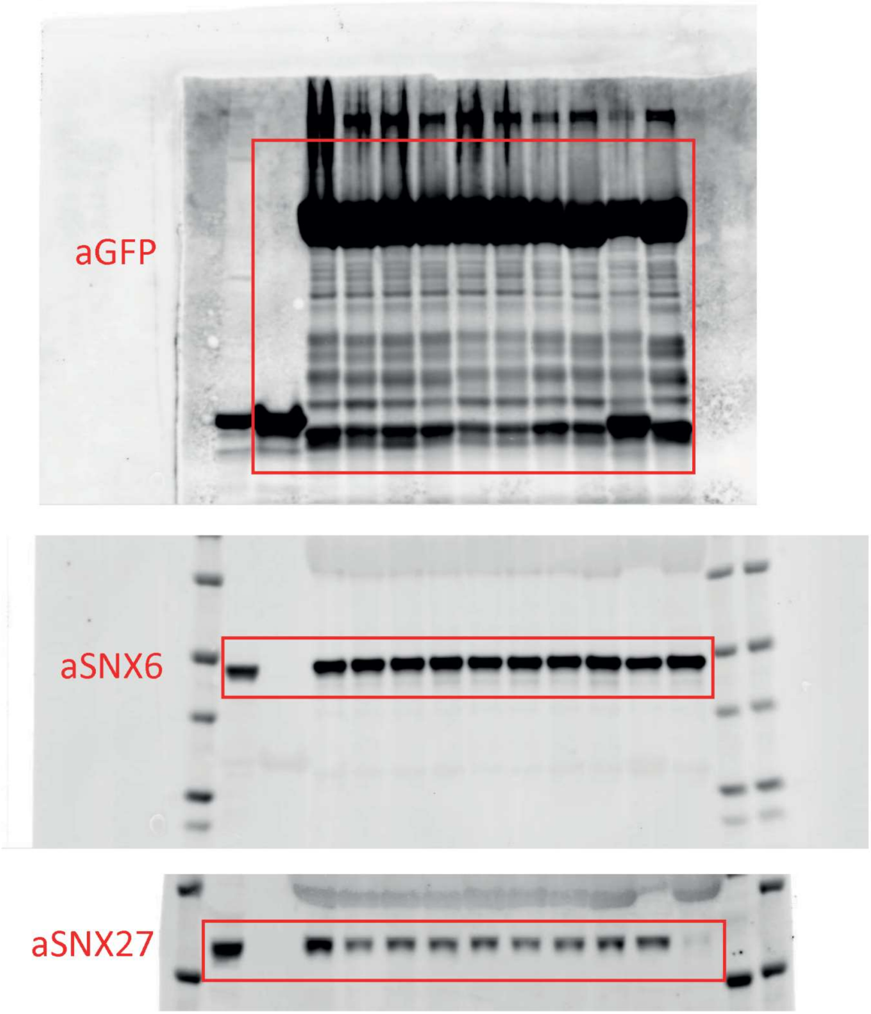

Figure 4B

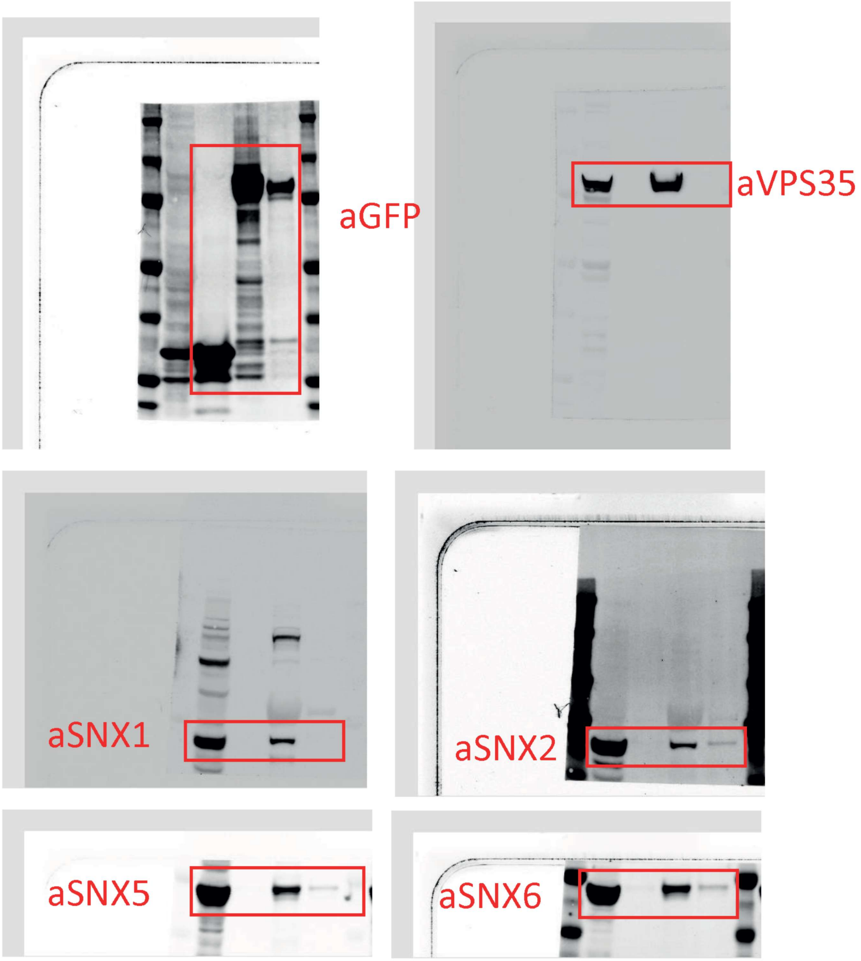

Figure 4D

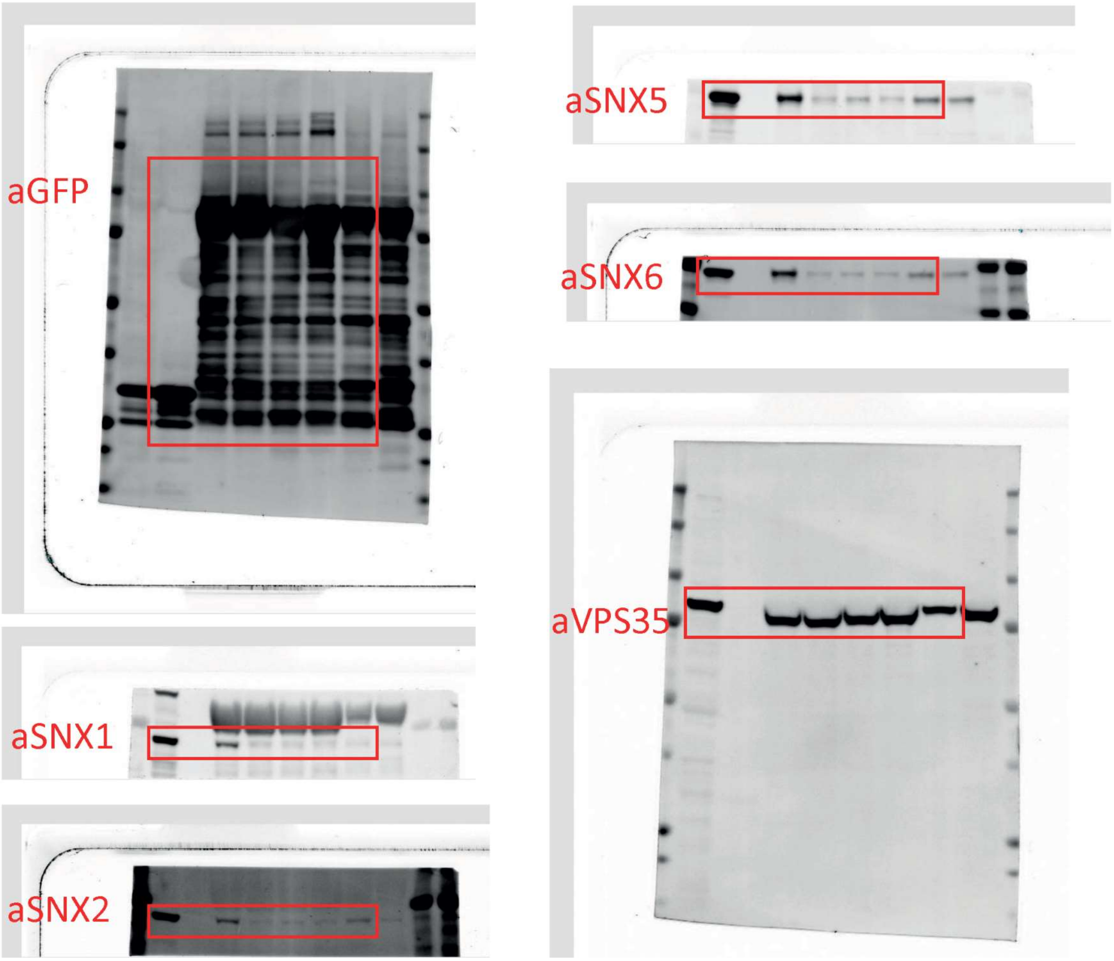

Figure 4G

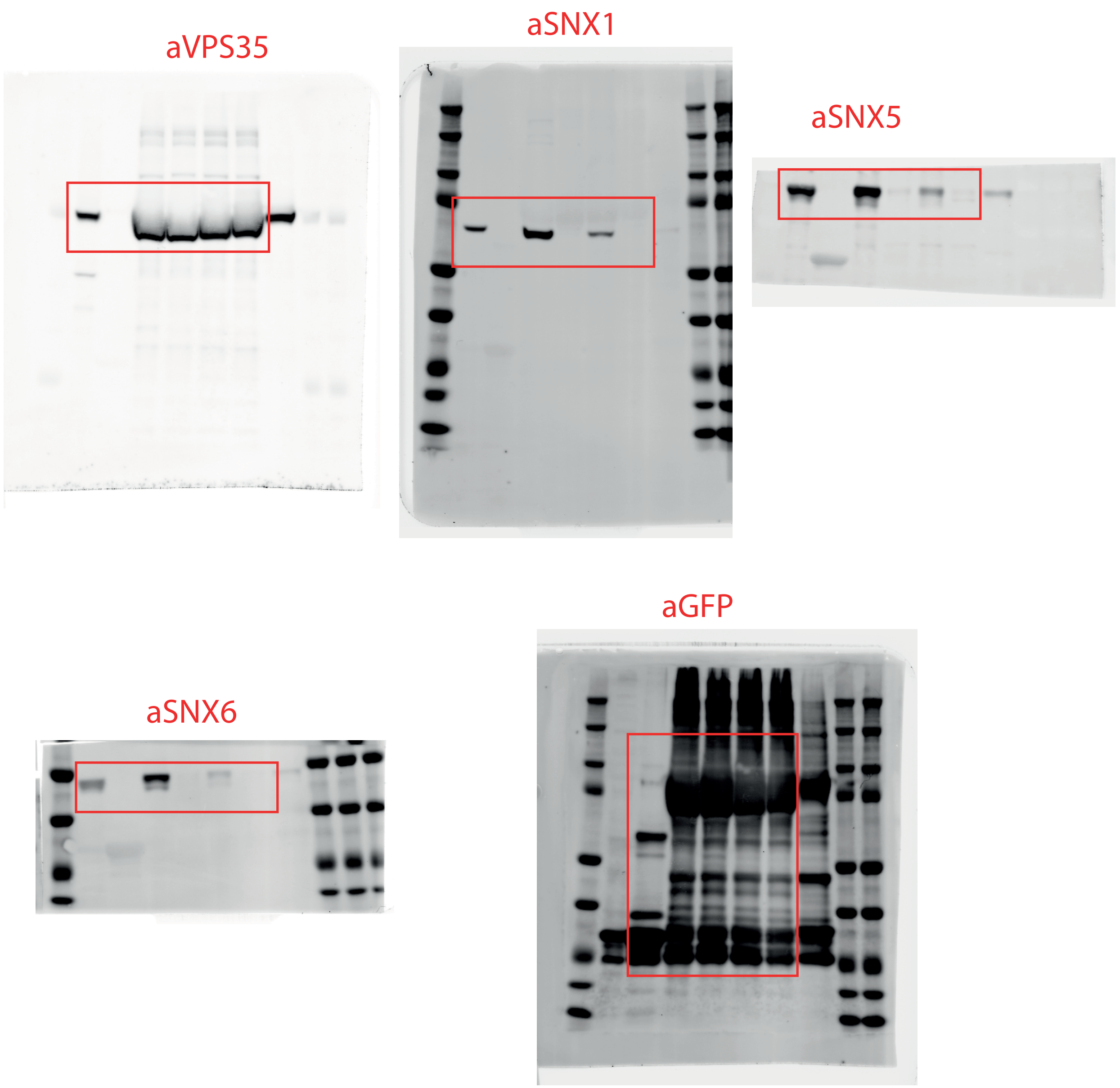

Figure 5A

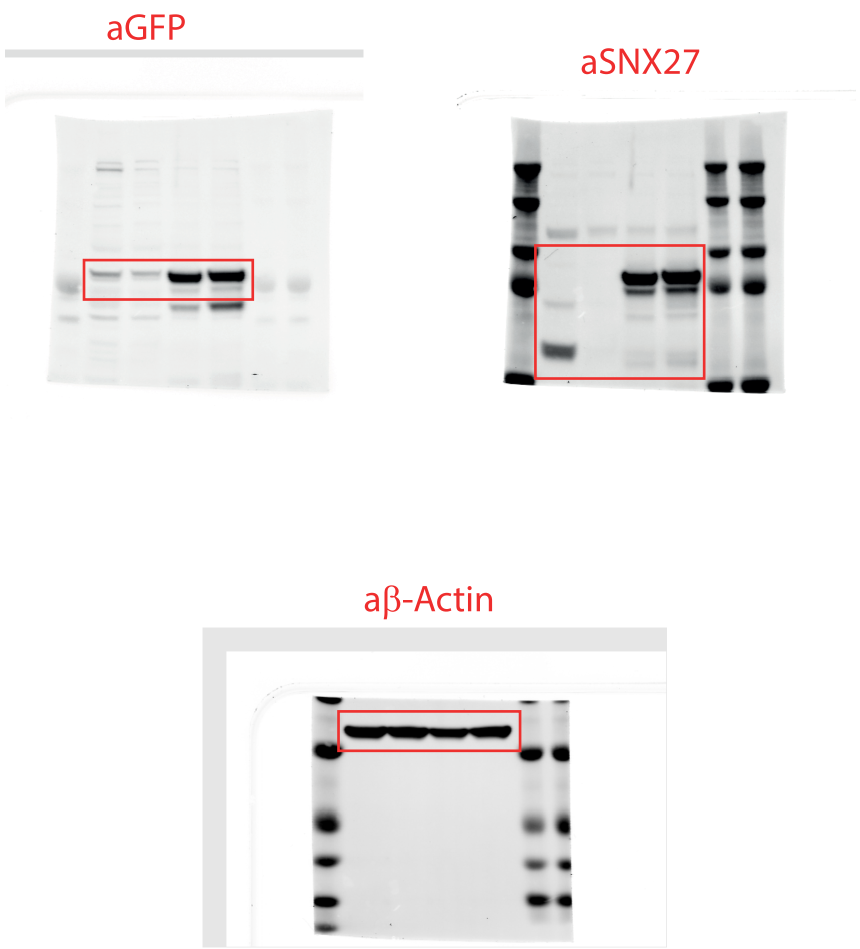

Figure 5C

Figure 5D

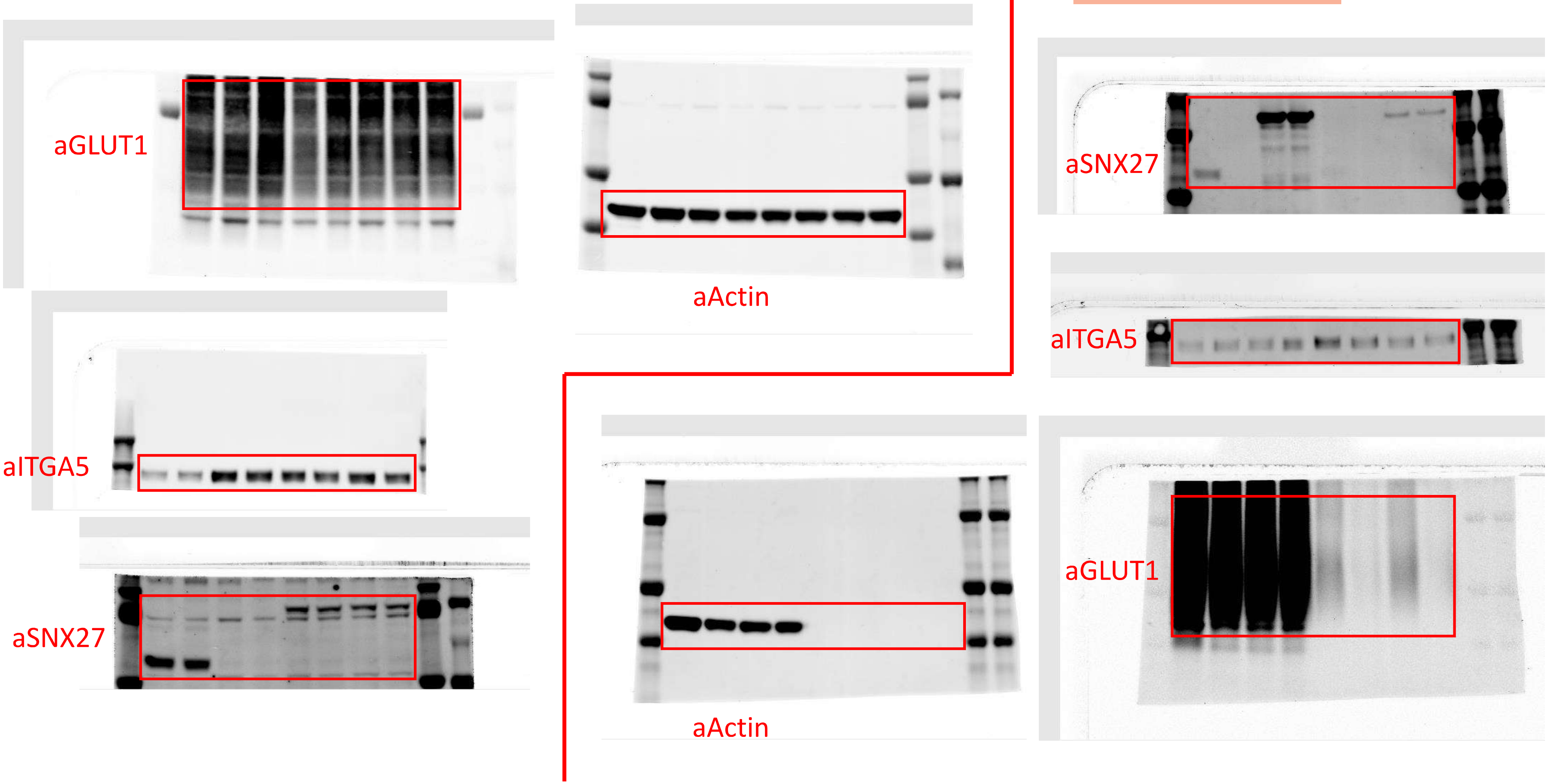

Figure 6A

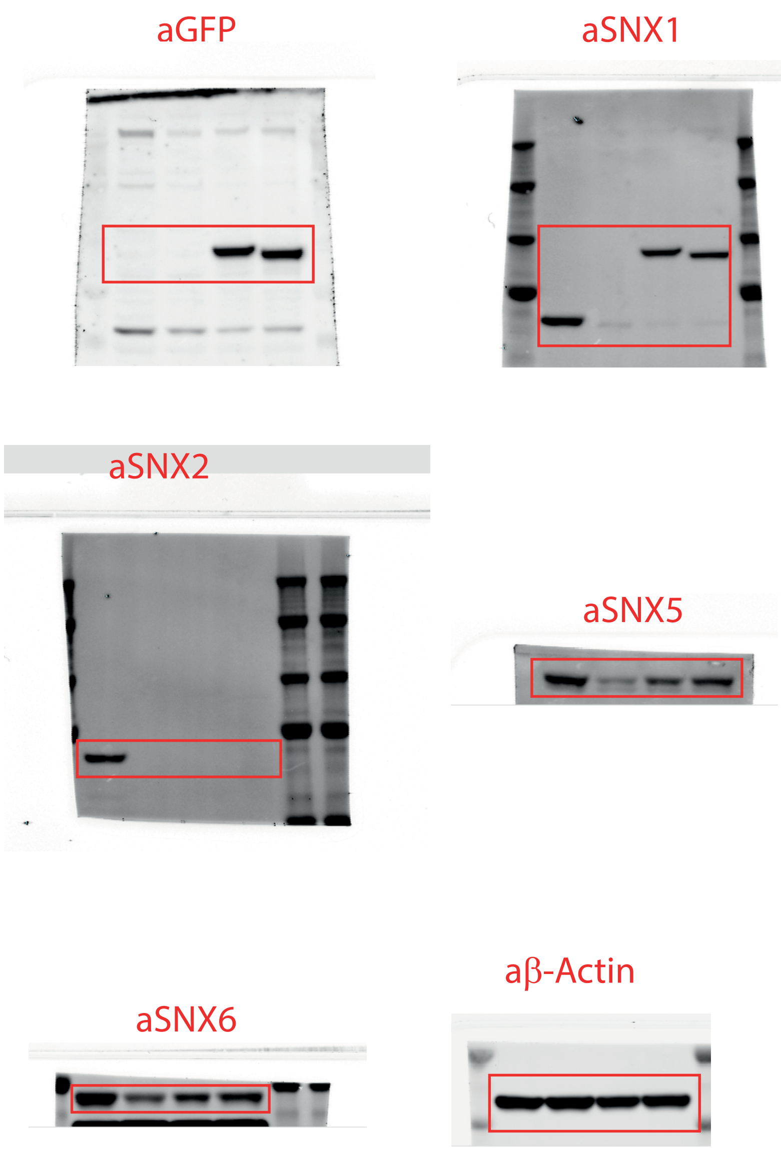

Figure 6C

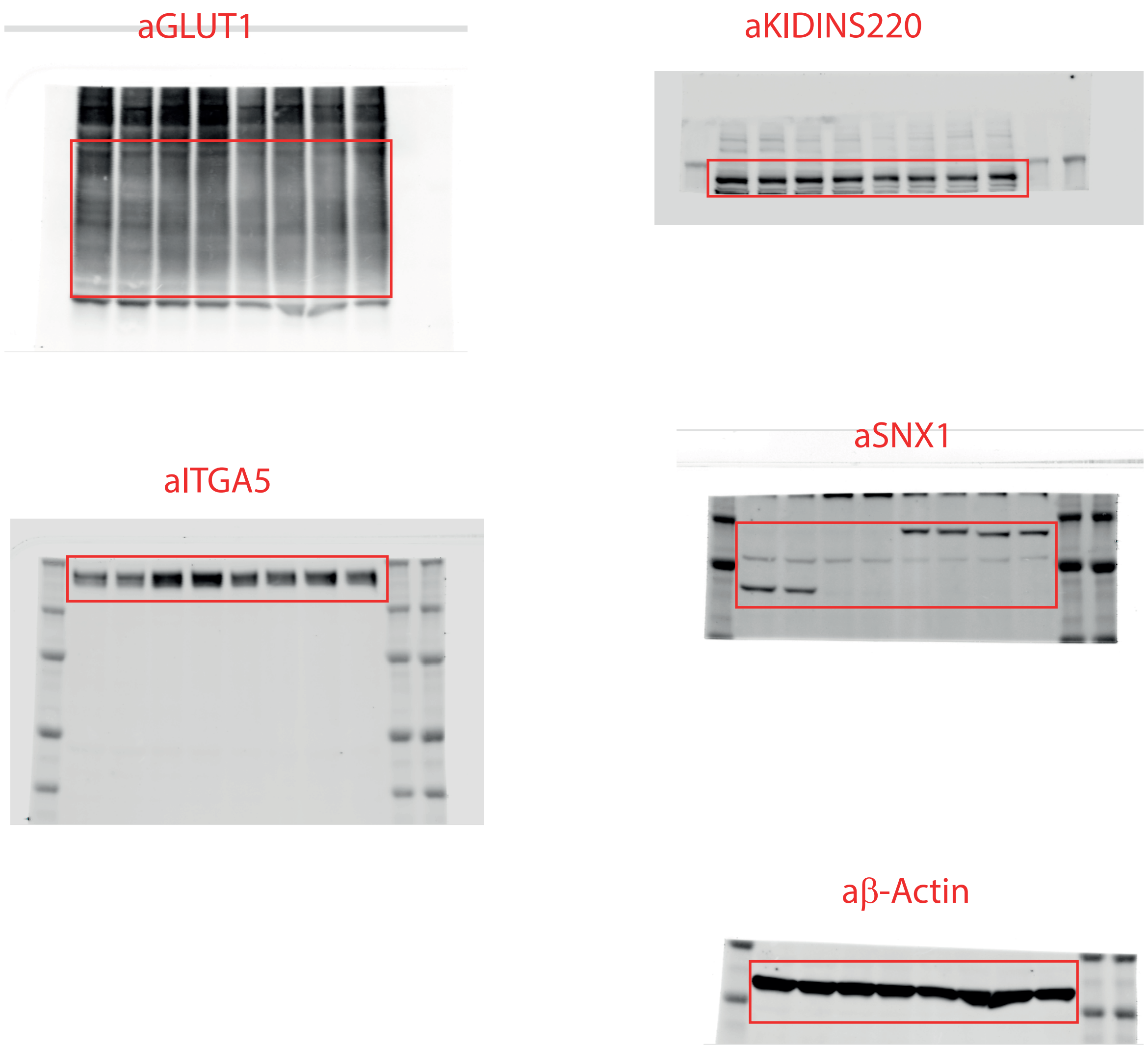

Figure 6D

aGLUT1

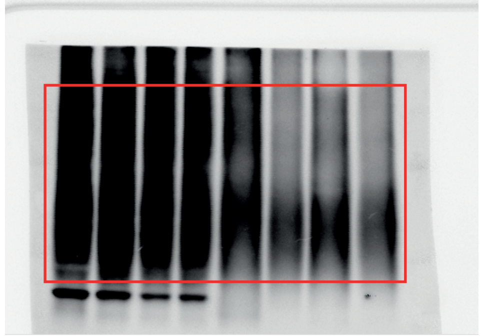

aITGA5

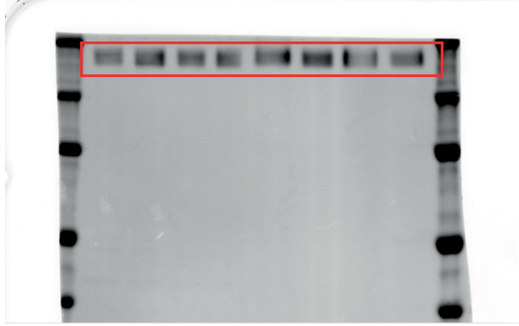

aSNX1

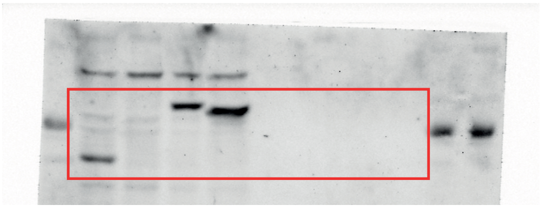

aβ-Actin

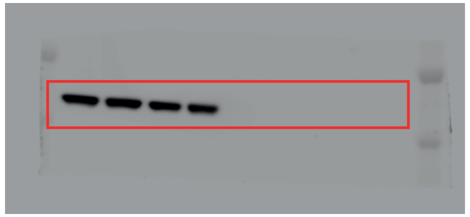

Supplement: S1 Raw Images — (PDF) [file pbio.3001601.s006.pdf]
